# Changes in Physical Activity Across Cancer Diagnosis and Treatment Based on Smartphone Step Count Data Linked to a Japanese Claims Database: Retrospective Cohort Study

**DOI:** 10.2196/58093

**Published:** 2025-01-20

**Authors:** Yoshihide Inayama, Ken Yamaguchi, Kayoko Mizuno, Sachiko Tanaka-Mizuno, Ayami Koike, Nozomi Higashiyama, Mana Taki, Koji Yamanoi, Ryusuke Murakami, Junzo Hamanishi, Satomi Yoshida, Masaki Mandai, Koji Kawakami

**Affiliations:** 1 Department of Gynecology and Obstetrics Graduate School of Medicine and Faculty of Medicine Kyoto University Kyoto Japan; 2 Department of Pharmacoepidemiology Graduate School of Medicine and Public Health Kyoto University Kyoto Japan; 3 Department of Digital Health and Epidemiology Graduate School of Medicine and Public Health Kyoto University Kyoto Japan; 4 Laboratory of Epidemiology and Prevention Kobe Pharmaceutical University Kobe Japan; 5 Institute of Medicine University of Tsukuba Tsukuba Japan

**Keywords:** cancer, lifelog data, physical activity, quality of life, step count, Japanese, database, smartphone, mobile app, exercise, mobile phone

## Abstract

**Background:**

Although physical activity (PA) is recommended for patients with cancer, changes in PA across cancer diagnosis and treatment have not been objectively evaluated.

**Objective:**

This study aimed to assess the impact of cancer diagnosis and treatment on PA levels.

**Methods:**

This was a retrospective cohort study using a Japanese claims database provided by DeSC Healthcare Inc, in which daily step count data, derived from smartphone pedometers, are linked to the claims data. In this study, we included patients newly diagnosed with cancer, along with those newly diagnosed with diabetes mellitus for reference. We collected data between April 2014 and September 2021 and analyzed them. The observation period spanned from 6 months before diagnosis to 12 months after diagnosis. We applied a generalized additive mixed model with a cubic spline to describe changes in step counts before and after diagnosis.

**Results:**

We analyzed the step count data of 326 patients with malignant solid tumors and 1388 patients with diabetes. Patients with cancer exhibited a 9.6% (95% CI 7.1%-12.1%; *P*<.001) reduction in step counts from baseline at the start of the diagnosis month, which further deepened to 12.4% (95% CI 9.5%-15.2%; *P*<.001) at 3 months and persisted at 7.1% (95% CI 4.2%-10.0%; *P*<.001) at 12 months, all relative to baseline. Conversely, in patients with diabetes, step counts remained relatively stable after diagnosis, with a slight upward trend, resulting in a change of +0.6% (95% CI –0.6% to 1.9%; *P*=.31) from baseline at 3 months after diagnosis. At 12 months after diagnosis, step counts remained decreased in the nonendoscopic subdiaphragmatic surgery group, with an 18.0% (95% CI 9.1%-26.2%; *P*<.001) reduction, whereas step counts returned to baseline in the laparoscopic surgery group (+0.3%, 95% CI –6.3% to 7.5%; *P*=.93).

**Conclusions:**

The analysis of objective pre- and postdiagnostic step count data provided fundamental information crucial for understanding changes in PA among patients with cancer. While cancer diagnosis and treatment reduced PA, the decline may have already started before diagnosis. The study findings may help tailor exercise recommendations based on lifelog data for patients with cancer in the future.

## Introduction

With the increasing number of cancer survivors, attention has been paid to not only cancer remission but also quality of life, which strongly correlates with physical activity (PA) in patients with cancer [1,2]. PA has been reported to be associated with a favorable prognosis in cancer survivors, with associations demonstrated in various cancer types, such as breast and colorectal cancers [1,3].

Although PA is a pivotal factor in patients with cancer, objective evidence on how it declines after cancer diagnosis and treatment is still lacking. Objective evaluation of prediagnostic PA is challenging. Previous studies that compared PA before and after cancer diagnosis relied on retrospective recall [4,5], which may introduce recall bias [6]. Therefore, research using objective precancer onset data is necessary to avoid this bias. However, this is challenging in studies that recruit participants after hospital visits.

Recently, many studies have leveraged real-world data collected during daily clinical practice and patients’ daily lives [7]. With advancements in digital devices, access to patients’ daily life data has improved [8]. Furthermore, the prevalent use of smartphone apps has facilitated the routine tracking of step counts, which strongly correlate with PA levels [9,10]. A daily step count of at least 10,000 steps is recommended for adults by the World Health Organization, while Japan’s Ministry of Health, Labour and Welfare sets a more realistic target of 8000 steps per day for adults [11,12]. Integrating step count data obtained using smartphones with medical information allows for objective evaluation of changes in PA from the prediagnosis period. Thus, this study aimed to assess the effect of cancer diagnosis and treatment on PA levels by analyzing real-world step count data.

## Methods

### Data Source

This was a retrospective cohort study analyzing a database provided by DeSC Healthcare Inc, in which daily step count data are linked to a Japanese claims database [13,14]. Briefly, members of affiliated health insurance associations can access the Kencom smartphone app, developed by DeSC Healthcare Inc, free of charge. Daily step counts were measured using the Kencom app, synchronized with smartphone pedometers [13,14]. The Kencom database was integrated and anonymized with the Japanese claims database in affiliated health insurance associations under the opt-out agreement. Subsequently, DeSC Healthcare Inc merged data from various insurance associations, creating a large, longitudinal database for research use [13,14]. Diseases were classified according to *International Classification of Diseases, 10th edition* (*ICD-10*) codes. This database, which integrates step count information with medical information, mainly includes employees of large businesses and their dependents [13,14]. In this study, step count data were used to quantify PA levels.

### Study Population

This study included patients newly diagnosed with malignant tumors (*ICD-10* code: C). In addition, patients newly diagnosed with diabetes mellitus (*ICD-10* code: E11-E14), a representative chronic disease in which the importance of PA is widely recognized [15], were included as a positive control, given the expectation that step counts would increase after diagnosis [16]. The inclusion of newly diagnosed patients also simplified the identification of index time [17]. Data between April 2014 and September 2021 were analyzed. The observation period spanned from 6 months before diagnosis to 12 months after diagnosis. The detailed eligibility criteria are described in Table S1 in Multimedia Appendix 1. Patients who did not receive treatment were excluded, and the Japanese Claims database has been validated as having over 98% specificity for identifying patients using this method [18].

### Statistical Analysis

We applied a generalized additive mixed model with a cubic spline to describe changes in step counts. Individual-specific random effects, age at diagnosis, seasonal effects, day of the week (weekday, weekend, or holiday), and sex were included as covariates. Days with missing step count records were excluded from the analysis. A negative binomial distribution was assumed for step counts because of the considerable variability.

Subgroup analysis was performed on changes in step counts stratified by treatment method and cancer type. The definitions of treatment methods and cancer types are described in Tables S2 and S3 in Multimedia Appendix 1, respectively. Statistical analyses including data cleaning were conducted using R version 4.3.1 (R Foundation) with packages *mgcv* (version 1.8-42) and *ggplot2* (version 3.4.2).

### Ethical Considerations

The Kyoto University Graduate School and Faculty of Medicine Ethics Committee approved this study (reference R3514). The need to obtain written informed consent from patients was waived because the data were anonymized.

## Results

### Study Population

A final cohort of 326 patients with malignant solid tumors and 1388 patients with diabetes met the eligibility criteria and were included in the analysis (Figure S1 in Multimedia Appendix 1). Table 1 shows patient characteristics and their respective cancer treatments. The median age of patients with cancer was 51 (IQR 45-56) years, and that of patients with diabetes was 50 (IQR 44-55) years. The median proportion of missing step count days during the observation period was 0.5% (IQR 0.0%-2.1%) among patients with cancer and 0.2% (IQR 0.0%-1.9%) in patients with diabetes. Further data on specific cancer diagnoses and the corresponding treatment for patients with cancer are described in Table S4 in Multimedia Appendix 1.

**Table 1:** Patient characteristics.

Characteristic		Patients with cancer (n=326)	Patients with diabetes mellitus (n=1388)	*P* values
**Age (years), median (IQR)**		51 (45-56)	50 (44-55)	.38
**Male sex, n (%)**		194 (59.5)	1144 (82.4)	<.001
**Surgeries, n (%)**		207 (63.5)	—^a^	—
**Small interventions^b^, n (%)**		114 (35)	—	—
**Radiation, n (%)**		67 (20.6)	—	—
**Conventional cytotoxic chemotherapies, n (%)**		67 (20.6)	—	—
**Molecular targeted therapy, n (%)**		16 (4.9)	—	—
**Immunotherapies, n (%)**		3 (0.9)	—	—
**Daily step counts for the reference month^c^, median (IQR)**		5754.9 (4219.2-7283.5)	6210.6 (4339.4-8015.8)	.01
**Year of diagnosis, n (%)**				
	2015	6 (1.8)	49 (3.5)	—
	2016	24 (7.4)	137 (9.9)	—
	2017	52 (16)	204 (14.7)	.44
	2018	81 (24.8)	324 (23.3)	—
	2019	102 (31.3)	414 (29.8)	—
	2020	61 (18.7)	260 (18.7)	—

^a^Not applicable

^b^The following procedures were classified as small interventions: cervical conization, diagnostic laparoscopy and thoracoscopy, endometrial curettage, excision of skin tumors, orchiectomy, transurethral resection of bladder tumors, and upper and lower gastrointestinal endoscopic surgeries (mucosal resection, polypectomy, and submucosal dissection).

^c^Reference month is month −1 for patients with cancer and month 0 for patients with diabetes mellitus (Figure 1).

**Figure 1:**
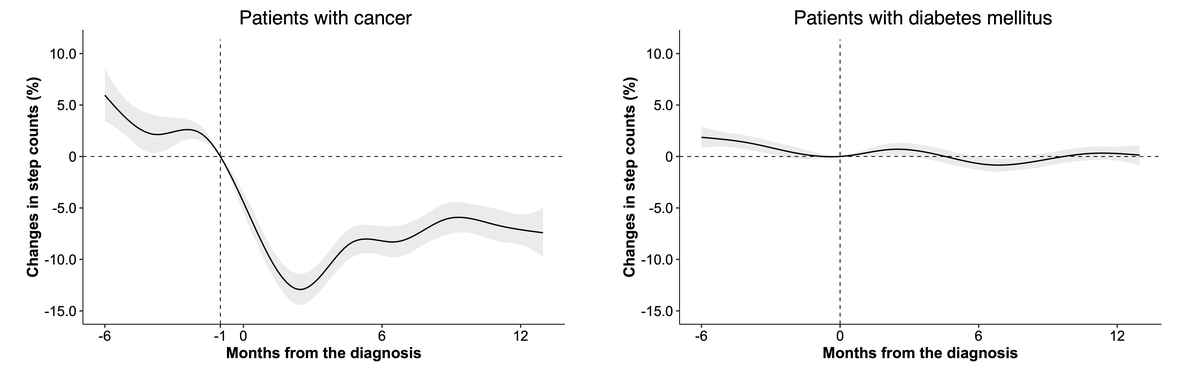
Estimated daily step count changes before and after diagnosis. Daily step count changes in (left) patients diagnosed with cancer and (right) patients diagnosed with diabetes mellitus are described. Shading indicates the SE. Month 0 signifies the month of diagnosis. For patients with cancer, the baseline for step count change was defined as the first day of month −1, accounting for the time needed for pathology after biopsy or diagnostic surgery to confirm diagnosis.

### Changes in Step Counts

The results of the descriptive analysis of step count data are presented in Figure S2 in Multimedia Appendix 1. Step counts showed no significant difference between patients with cancer and those with diabetes 2 months before diagnosis (*P*=.17) but declined significantly in the former during the diagnosis month (*P*<.001). In subsequent analyses, the baseline for step counts in patients with cancer was therefore set at the beginning of the month before diagnosis. Figure 1 shows the percent change in step counts over time in relation to the time of diagnosis. Patients with cancer showed a 9.6% (95% CI 7.1%-12.1%; *P*<.001) reduction in step counts from baseline in the month of diagnosis, which deepened to 12.4% (95% CI 9.5%-15.2%; *P*<.001) at 3 months and persisted at 7.1% (95% CI 4.2%-10.0%; *P*<.001) at 12 months, all relative to baseline. Conversely, in patients with diabetes, step counts remained relatively stable after diagnosis, with a slight upward trend, resulting in a change of +0.6% (95% CI –0.6% to 1.9%; *P*=.31) from baseline at 3 months after diagnosis. The change from baseline at 12 months after diagnosis was not significant (+0.3%, 95% CI –1% to 1.6%; *P*=.68).

### Changes in Step Counts According to Treatment Modalities

**Figure 2:**
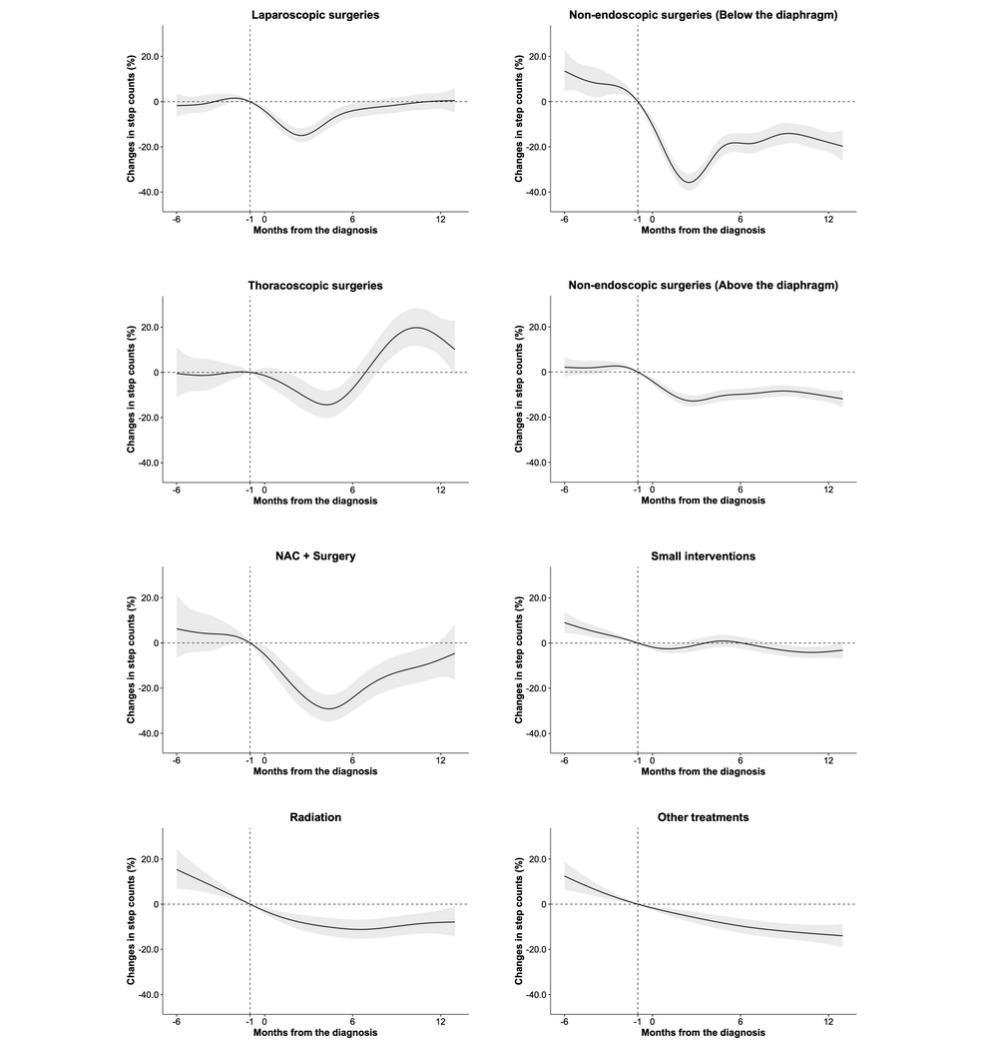
Estimated daily step count changes before and after diagnosis by treatment methods: Other treatments include conventional cytotoxic chemotherapy, immunotherapy, molecular targeted therapy, small interventions with chemotherapy or immunotherapy, and chemoradiotherapy. Shading indicates the SE. NAC: neoadjuvant chemotherapy.

Figure 2 shows the results of the subgroup analysis stratified by treatment modalities. Patients who received systemic chemotherapy without surgery were classified as the other treatment group because of the limited number of patients. There were no significant changes in the distribution of patient numbers by treatment group across the years (*P*=.69; Table S5 in Multimedia Appendix 1). At 3 months after diagnosis, the reduction in step counts from baseline was 14.3% (95% CI 8.0%-20.1%; *P*<.001) in the laparoscopic surgery group and 34.2% (95% CI 26.6%-41.1%; *P*<.001) in the nonendoscopic subdiaphragmatic surgery group. At 12 months after diagnosis, step counts remained decreased in the nonendoscopic subdiaphragmatic surgery group, with an 18.0% (95% CI 9.1%-26.2%; *P*<.001) reduction, whereas step counts returned to baseline in the laparoscopic surgery group (+0.3%, 95% CI –6.3% to 7.5%; *P*=.93). At 12 months after diagnosis, the nonendoscopic supradiaphragmatic surgery group showed a 10.9% (95% CI 5.7%-15.7%; *P*<.001) step count reduction, whereas this reduction was not seen in the thoracoscopic surgery group, and the step count change was not significant (+15.2%, 95% CI –0.4% to 33.1%; *P*=.06).

### Changes in Step Counts According to Cancer Type

Figure S3 and Table S6 in Multimedia Appendix 1 show the results of the subgroup analysis stratified by cancer type. A significant postoperative reduction in step counts was observed in patients with gynecologic cancers, and the majority of them underwent nonendoscopic surgeries (endometrial cancer: 4/5, 80%; cervical cancer: 3/6, 50%; and ovarian cancer: 3/4, 75%). Conversely, in gastrointestinal and urologic cancers, where abdominal surgeries were also performed, the proportion of patients who underwent small interventions or laparoscopic surgeries was high, and the postoperative reduction in step counts was not as pronounced.

## Discussion

### Principal Findings

The analysis of objective pre- and postdiagnostic lifelog data showed that step counts in patients with cancer decreased with cancer diagnosis and treatment, with step counts already below baseline at the beginning of the diagnosis month and failing to return to baseline 12 months later. Conversely, the change from baseline in those newly diagnosed with diabetes was not significant. The decline in step count varies with treatment and cancer type.

### Comparison With Previous Work

To the best of our knowledge, this is the first study to objectively describe the decline in PA levels from precancer diagnosis using lifelog data on step counts. Previous studies that relied on retrospective recall have reported a decline in PA by 59% after treatment of breast cancer [4]. In colorectal cancer, the reduced PA during treatment improved after treatment completion [5]. Our analysis using objective real-world data provides a closer representation of real-life events. In patients with diabetes, while the increase in step counts was not significant, an upward trend was observed after diagnosis, consistent with previous reports [16].

This study showed possible variations in PA measured as step count based on the specific treatment modality used. Treatments for specific cancer types are typically standardized. However, by analyzing patients with varying cancer types, this study explored the impacts of different treatments on step counts. Interestingly, a prominent decrease in step counts was observed in patients who underwent nonendoscopic surgery, suggesting that opting for less invasive surgical approaches may help mitigate the decrease in step counts. However, this result should be carefully interpreted because patients requiring open surgery may have had more advanced stages with worse prognoses or require more intensive treatment. Further examination is needed to assess the long-term impact of surgical approaches on PA.

Measuring PA has become increasingly accessible through the use of smartphones and other wearable devices [8]. Previous studies have been conducted to categorize PA in patients with cancer using these devices. However, no established threshold has been identified [19]. This study adds new insights by focusing on changes in PA, which are recorded as step counts. Step counts serve as a valid and useful surrogate marker of PA, and a decrease in step counts is strongly associated with an increase in all-cause mortality [20,21]. Therefore, conducting close follow-ups on posttreatment PA levels using step counts has the potential to contribute to patient-centered care and personalized recommendations for PA, which remain insufficient [2]. Further studies are needed to address tailoring recommendations for PA during cancer treatment according to cancer type, treatment approach, and comorbidities.

### Limitations

This study has some limitations. First, influences beyond cancer diagnosis and treatment, such as aging, societal changes, and changes in smartphone pedometer specifications due to device upgrades or replacements by users, may be involved. However, minimal changes in step counts in the diabetes group indicated a limited impact of these factors. Second, the proportion of patients managed with small interventions was high, indicating that a considerable number of patients were detected at an early stage. This might be because this study included relatively young patients with a median age of 51 (IQR 45-56) years, primarily employees of large businesses and their dependents, who were exclusively Kencom app users, indicative of a potential high health awareness demographic. Therefore, this population may not represent the general Japanese population. However, the decline in step count levels would be more pronounced in patients with a higher prevalence of advanced-stage cancer, who are likely to undergo more invasive treatments [22]. Third, the cancer stage was not determined in this study. However, because treatments are generally determined by cancer stage, results stratified by treatment may have higher generalizability to populations with different cancer stages. Fourth, the follow-up period in this study was 1 year, which did not allow for the analysis of step count data in relation to patient prognosis. PA may improve prognosis in cancer survivors [1,3]. Therefore, further studies are needed to assess its effect on patient prognosis. Finally, step count data may not fully reflect actual PA levels, as its accuracy can be affected by factors, including the possibility that individuals are not always carrying their smartphones.

### Conclusions

This study used objective pre- and postdiagnostic step count data and showed that while PA decreased with cancer diagnosis and treatment, the decline may have already started before diagnosis. The decline varies with treatment and cancer type, with a notable decrease in patients who underwent nonlaparoscopic abdominal surgeries. Assessing PA using step count data has the potential to contribute to patient-centered care. This finding may help tailor exercise recommendations based on lifelog data for patients with cancer.

## Appendix

Multimedia Appendix 1 Eligibility criteria, definition of subgroups by treatment method, definition of subgroups by cancer type, patient characteristics by cancer type, the annual number of patients in each treatment group, summary of step count changes by cancer type, selection of study samples, distribution of mean daily step counts in each month, and estimated daily step count changes before and after diagnosis by cancer type.

## Abbreviations

ICD-10: International Classification of Diseases, 10th edition
PA: physical activity

## Data Availability

The datasets analyzed during this study are not publicly available due to the contract with the data provider.
