# Supplementary material for: Changes in Physical Activity Across Cancer Diagnosis and Treatment Based on Smartphone Step Count Data Linked to a Japanese Claims Database: Retrospective Cohort Study
Source: JMIR Cancer. 2025 Jan 20;11:e58093. doi: 10.2196/58093 (PMC11791449; doi:10.2196/58093)

Multimedia Appendix 1.

Table S1. Eligibility criteria

Table S2. Definition of subgroups by treatment method

Table S3. Definition of subgroups by cancer type

Table S4. Patient characteristics by cancer type

Table S5. The annual number of patients in each treatment group

Table S6. Summary of step count changes by cancer type

Figure S1. Selection of the study samples

Figure S2. Distribution of mean daily step counts in each month

Figure S3. Estimated daily step count changes before and after diagnosis by cancer type

Table S1. Eligibility criteria

Inclusion criteria	Patients newly diagnosed with malignant tumors (ICD-10 code: C; cancer patients), and those with diabetes mellitus (ICD-10 code: E11–E14; control group)
Exclusion criteria (both groups)	Those with a look-back period of less than 6 months
	Those who did not initiate step recording 6 months before diagnosis
	Those who did not continuously record step data by the end of the observation period[a]
	Those had data for fewer than 80% of the observation days
Exclusion criteria for cancer patients	Those with nonsolid tumors
	Those with recurrent cancer
	Those receiving treatment for multiple malignant tumors during the observation period[a]
	Those who did not receive treatment for malignant tumors during the observation period[a]
Exclusion criteria for diabetes patients	Individuals who did not receive guidance or treatment for diabetes mellitus during the observation period[a]

[a]From 6 months before diagnosis to 12 months after.

Table S2. Definition of subgroups by treatment method

Subgroups	Definition
Nonendoscopic surgery above the diaphragm<br>Nonendoscopic surgery below the diaphragm<br>Thoracoscopic surgery[a]<br>Laparoscopic surgery	Patients undergoing the specified surgery without chemotherapy and those with postoperative adjuvant chemotherapy[b,c]
NAC + surgery	Patients who underwent surgery other than small interventions following NAC[c]
Small interventions	Patients who underwent small interventions[d] without receiving conventional chemotherapy, immune checkpoint inhibitors (ICI), targeted molecular therapy, or radiation therapy
Radiation	Patients who received radiation therapy but did not undergo surgery or chemotherapy.

[a]Including mediastinoscopy and endoscopic thyroid surgeries.

[b]No patients underwent multiple surgeries belonging to different categories.

[c]These groups included patients who underwent small interventions in addition to surgeries.

[d]The following procedures were classified as small interventions: cervical conization, diagnostic laparoscopy and thoracoscopy, endometrial curettage, excision of skin tumors, orchiectomy, transurethral resection of bladder tumors, and upper and lower gastrointestinal endoscopic surgeries (mucosal resection, polypectomy, and submucosal dissection).

NAC, neoadjuvant chemotherapy

Table S3. Definition of subgroups by cancer type

Cancer types	Definition by ICD-10 code
Breast	C50
Ovarian	C56, C570
Cervical	C53
Endometrial	C54
Esophageal	C15
Gastric	C16
Colon	C18-20
Liver	C22
Bile	C248
Gallbladder	C23
Lung	C33-34
Bladder	C67
Prostate	C61
Renal	C64
Testis	C62
Thyroid	C73
Head and neck	C00-14, C30-32, C750, C760
Melanoma	C43
Other Skin	C44

ICD-10, the International Classification of Diseases, 10th edition

Table S4. Patient characteristics by cancer type

Cancer types	Breast (n = 71)	Endometrial (n = 5)	Cervical (n = 6)	Ovarian (n = 4)	Esophageal (n = 7)	Gastric (n = 26)	Colon (n = 70)	Liver (n = 3)	Gallbladder (n = 4)	Lung (n = 17)
Age (median [IQR])	47.0 [43.0, 52.0]	50.0 [49.0, 55.0]	44.0 [38.2, 48.2]	37.0 [34.5, 41.2]	57.0 [54.5, 60.0]	53.5 [47.0, 57.0]	49.0 [46.0, 54.8]	48.0 [46.5, 54.0]	46.5 [45.5, 47.8]	59.0 [51.0, 63.0]
Male (%)	0 (0.0)	0 (0.0)	0 (0.0)	0 (0.0)	6 (85.7)	22 (84.6)	57 (81.4)	3 (100.0)	4 (100.0)	15 (88.2)
Surgeries (%)	69 (97.2)	5 (100.0)	3 (50.0)	4 (100.0)	1 (14.3)	12 (46.2)	29 (41.4)	3 (100.0)	4 (100.0)	14 (82.4)
Small interventions (%)	0 (0.0)	3 (60.0)	2 (33.3)	0 (0.0)	6 (85.7)	16 (61.5)	51 (72.9)	0 (0.0)	0 (0.0)	0 (0.0)
Radiation (%)	42 (59.2)	0 (0.0)	1 (16.7)	0 (0.0)	0 (0.0)	1 (3.8)	1 (1.4)	1 (33.3)	0 (0.0)	2 (11.8)
Conventional cytotoxic chemotherapies (%)	26 (36.6)	1 (20.0)	0 (0.0)	1 (25.0)	0 (0.0)	3 (11.5)	17 (24.3)	1 (33.3)	1 (25.0)	3 (17.6)
Molecular targeted therapy (%)	11 (15.5)	0 (0.0)	0 (0.0)	0 (0.0)	1 (14.3)	0 (0.0)	1 (1.4)	0 (0.0)	0 (0.0)	2 (11.8)
Immunotherapies (%)	0 (0.0)	0 (0.0)	0 (0.0)	0 (0.0)	0 (0.0)	0 (0.0)	0 (0.0)	0 (0.0)	0 (0.0)	1 (5.9)

Cancer types	Melanoma (n = 3)	Other skin (n = 7)	Bladder (n = 13)	Renal (n = 13)	Prostate (n = 31)	Testicular (n = 6)	Thyroid (n = 14)	Head neck (n = 6)	Sarcoma (n = 8)	Others (n = 12)
Age (median [IQR])	33.0 [31.5, 44.0]	50.0 [48.5, 55.0]	58.0 [55.0, 64.0]	51.0 [48.0, 53.0]	60.0 [56.0, 63.0]	37.5 [31.0, 46.2]	45.0 [38.2, 49.8]	54.0 [51.5, 55.8]	47.5 [40.8, 48.8]	48.0 [39.2, 55.8]
Male (%)	2 (66.7)	5 (71.4)	13 (100.0)	9 (69.2)	31 (100.0)	6 (100.0)	3 (21.4)	6 (100.0)	4 (50.0)	8 (66.7)
Surgeries (%)	0 (0.0)	0 (0.0)	0 (0.0)	13 (100.0)	17 (54.8)	0 (0.0)	14 (100.0)	5 (83.3)	6 (75.0)	8 (66.7)
Small interventions (%)	3 (100.0)	7 (100.0)	13 (100.0)	0 (0.0)	0 (0.0)	6 (100.0)	0 (0.0)	0 (0.0)	3 (37.5)	4 (33.3)
Radiation (%)	0 (0.0)	0 (0.0)	0 (0.0)	0 (0.0)	15 (48.4)	0 (0.0)	0 (0.0)	1 (16.7)	1 (12.5)	2 (16.7)
Conventional cytotoxic chemotherapies (%)	0 (0.0)	0 (0.0)	10 (76.9)	0 (0.0)	0 (0.0)	0 (0.0)	0 (0.0)	0 (0.0)	1 (12.5)	3 (25.0)
Molecular targeted therapy (%)	0 (0.0)	0 (0.0)	0 (0.0)	0 (0.0)	0 (0.0)	0 (0.0)	0 (0.0)	0 (0.0)	0 (0.0)	1 (8.3)
Immunotherapies (%)	2 (66.7)	0 (0.0)	0 (0.0)	0 (0.0)	0 (0.0)	0 (0.0)	0 (0.0)	0 (0.0)	0 (0.0)	0 (0.0)

Table S5. The annual number of patients in each treatment group

	2015	2016	2017	2018	2019	2020
Laparoscopic surgeries	0	3	15	17	20	9
Non-endoscopic surgeries (Below the diaphragm)	1	1	5	10	8	4
Thoracoscopic surgeries	0	0	2	4	3	4
Non-endoscopic surgeries (Above the diaphragm)	3	9	17	18	26	19
NAC + Surgery	0	0	2	3	3	1
Small interventions	2	10	8	19	31	14
Radiation	0	0	0	7	5	4
Other treatments	0	1	3	3	6	6

P = .69 (Pearson's Chi-squared test with simulated *p-value*)

NAC, neoadjuvant chemotherapy

Table S6. Summary of step count changes by cancer type

Cancer type	Maximum reduction in step count (% [95% CI])	The time of maximum step count reduction (months)	*P* value
Bladder[a]	-4.1 [-13.4, 6.2]	-2.7	.42
Breast	-12.7 [-17.9, -7.2]	3.2	< .001
Cervical	-35.1 [-48.2, -18.6]	2.6	< .001
Colon	-10.5 [-16.2, -4.3]	6.8	.001
Endometrial	-27.9 [-42.5, -9.7]	2.6	.004
Esophageal	-12.9 [-26.1, 2.7]	2.8	.10
Gallbladder	-4.0 [-14.6, 8.0]	1.5	.50
Gastric	-17.7 [-26.4, -8.1]	2.4	.001
Head and neck	-13.8 [-26.3, 0.9]	1.1	.07
Liver	-1.9 [-27.3, 32.3]	12.9	.90
Lung	-13.1 [-23.1, -1.7]	3.6	.03
Malignant melanoma	-20.2 [-37.8, 2.5]	2.4	.08
Other skin cancers	-14.4 [-33.7, 10.4]	12.9	.23
Ovarian	-30.6 [-46.0, -10.7]	2.0	.004
Prostate	-9.7 [-16.7, -2.1]	4.9	.01
Renal	-15.7 [-26.9, -2.8]	2.2	.02
Sarcomas	-18.0 [-29.6, -4.5]	9.3	.01
Testicular	-1.4 [-8.7, 6.5]	3.6	.72
Thyroid	-13.6 [-26.3, 1.2]	12.9	.07
Other cancers	-32.0 [-41.9, -20.4]	3.2	< .001

[a]In bladder cancer patients, step count increased following diagnosis.

Figure S1. Selection of the study samples

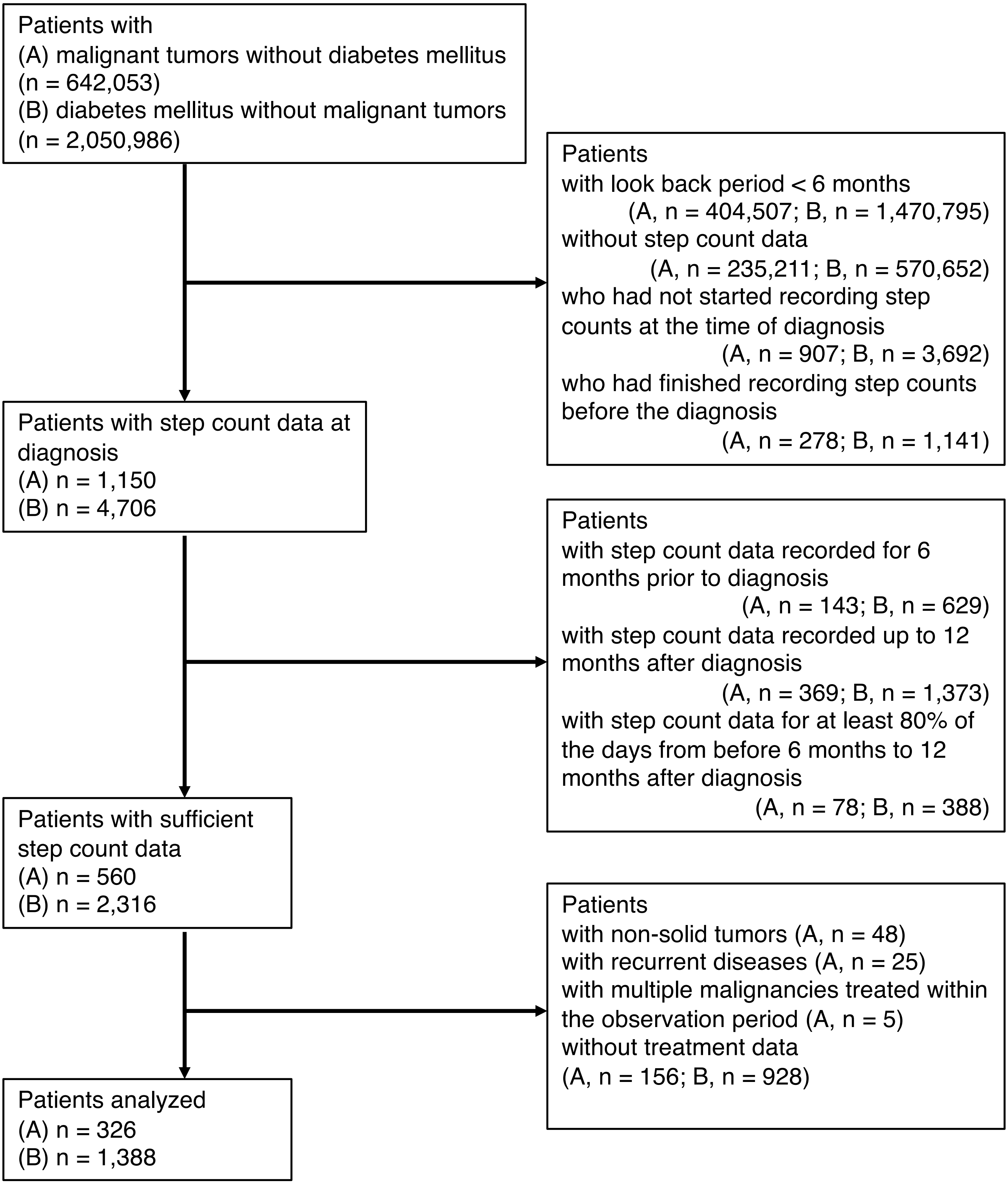

Figure S2.
Distribution of mean daily step counts in each month

The distribution of mean daily step counts for each patient is presented.
Bars indicate IQR.
IQR, interquartile range; DM, diabetes mellitus

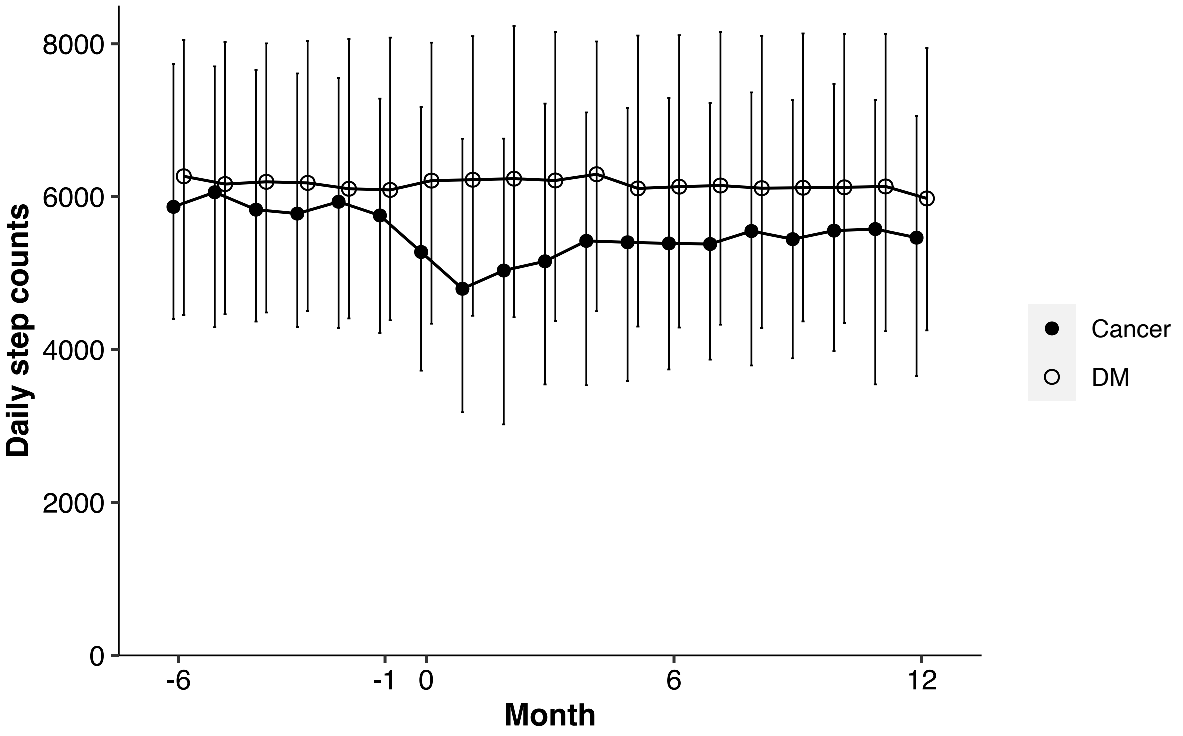

Month	Daily step counts for each month, median [IQR] Cancer	DM	*P* value
-6	5867.9 [4400.3, 7734.3]	6267.3 [4452.2, 8051.3]	0.14
-2	5934.3 [4284.7, 7553.2]	6104.2 [4407.8, 8062.5]	0.17
-1	5754.9 [4219.2, 7283.5]	6089.0 [4383.2, 8081.6]	0.016
0	5277.1 [3725.4, 7171.7]	6210.6 [4339.4, 8015.8]	<0.001
1	4796.5 [3180.6, 6758.9]	6222.4 [4443.3, 8099.8]	<0.001
2	5034.6 [3021.8, 6760.3]	6236.2 [4422.3, 8233.6]	<0.001
3	5155.4 [3544.5, 7218.7]	6212.6 [4375.1, 8154.3]	<0.001
6	5388.9 [3740.4, 7292.1]	6131.6 [4289.0, 8112.8]	<0.001
12	5464.6 [3652.6, 7056.9]	5977.5 [4251.3, 7944.4]	<0.001

Figure S3.
Estimated daily step count changes before and after diagnosis by cancer type

A notable postoperative reduction in step counts was noted among gynecological cancer patients, the majority of whom underwent non-endoscopic surgeries (endometrial cancer: 80% [4/5], cervical cancer: 50% [3/6], and ovarian cancer: 75% [3/4]). Conversely, in gastrointestinal and urologic cancers, where abdominal surgery is also performed, the proportion of patients who underwent small interventions or laparoscopic surgery was high, and the postoperative reduction in step counts was not as pronounced.
Shading indicates the standard error.
Other treatments include conventional chemotherapy, immunotherapy, molecular-targeted therapy, small interventions with chemotherapy or immunotherapy, and chemoradiation therapy.
NAC, neoadjuvant chemotherapy.

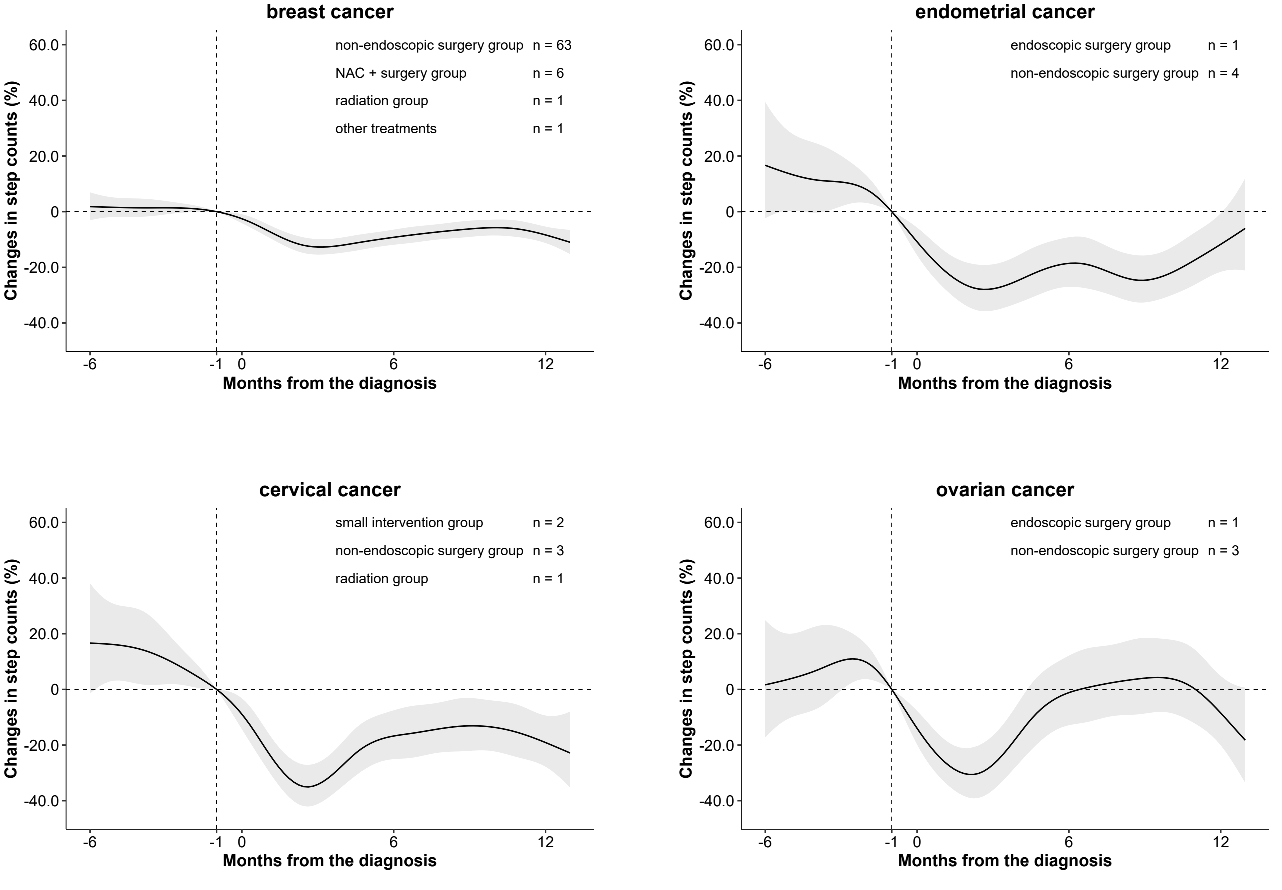

Figure S3. (Continued)

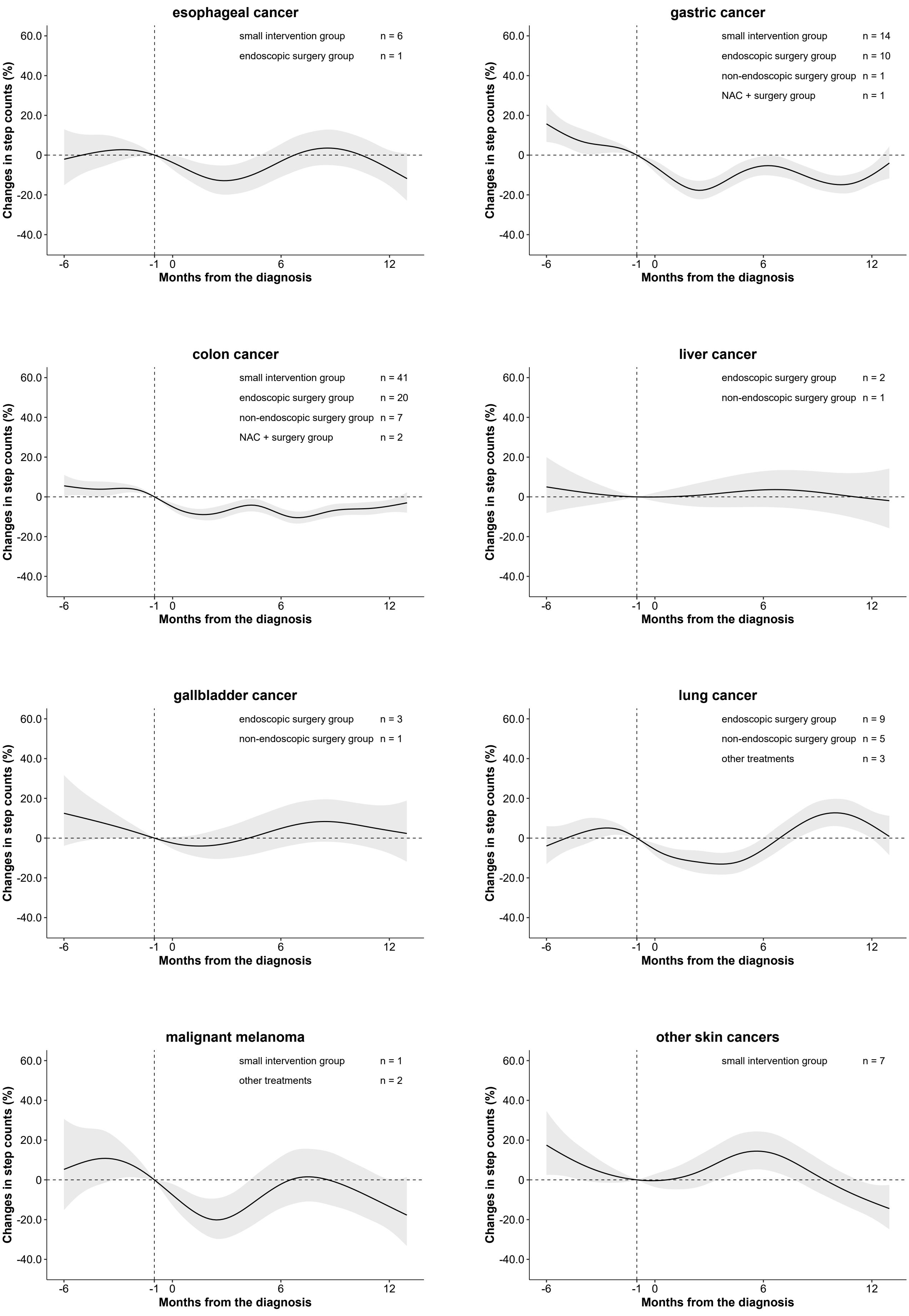

Figure S3. (Continued)

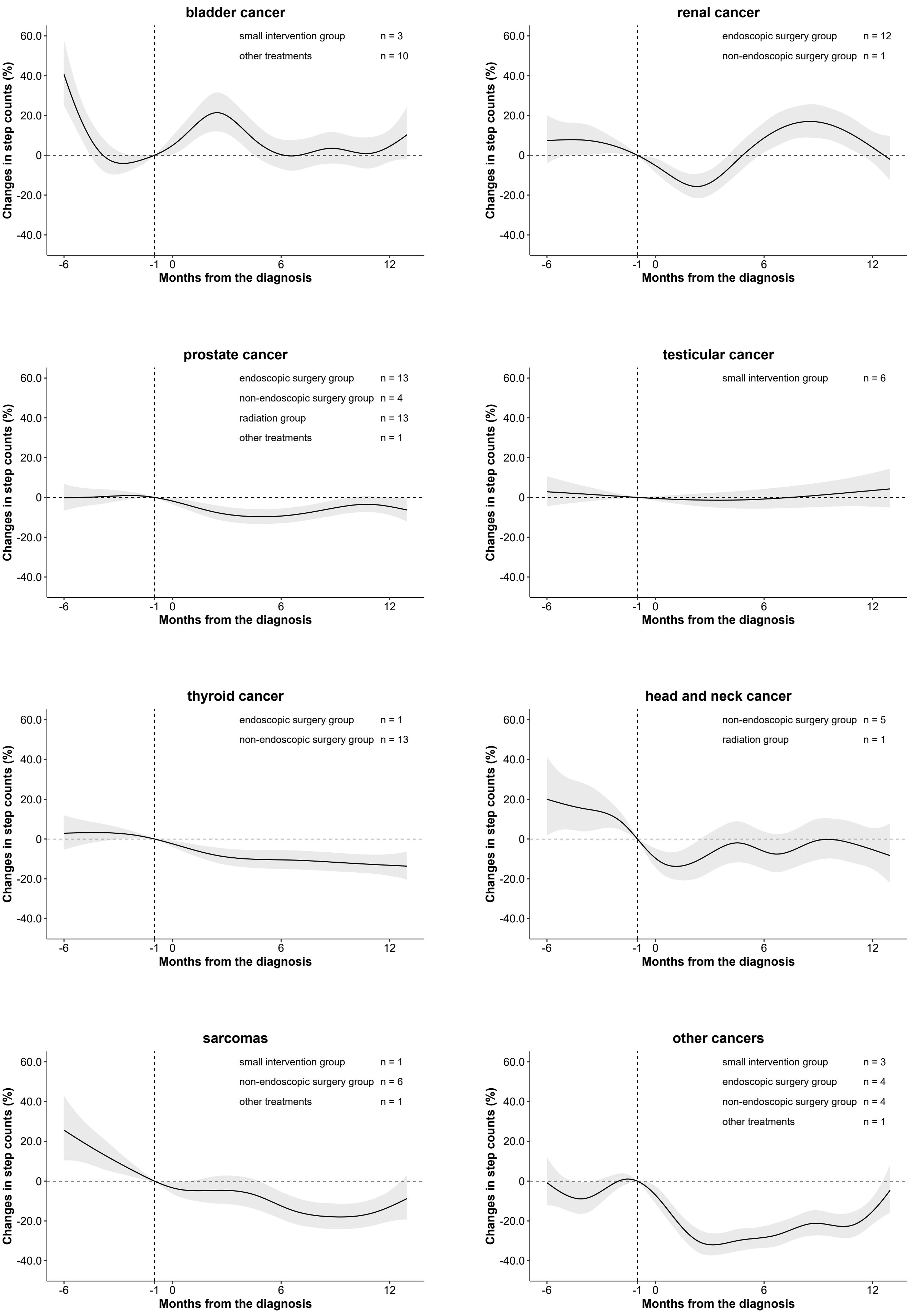

Supplement: Multimedia Appendix 1 [file cancer_v11i1e58093_app1.pdf]
